# MRI-based clinicoradiologic model for identifying MT-HCC with distinct postoperative prognosis and potential association with postoperative TKI-ICI therapy

**DOI:** 10.3389/fimmu.2026.1806601

**Published:** 2026-05-08

**Authors:** Yiman Li, Zhebo Qin, Jie Cheng, Xiaofeng Li, Chen Liu, Qingrui Li, Huarong Zhang, Ping Cai, Fengxi Chen, Wei Chen, Xiaoming Li

**Affiliations:** 1 7T Magnetic Resonance Translational Medicine Research Center, Department of Radiology, Southwest Hospital, Army Medical University (Third Military Medical University), Chongqing, China; 2 Department of Ultrasound, Southwest Hospital, Army Medical University (Third Military Medical University), Chongqing, China; 3 Department of Radiology, The Third Affiliated Hospital of Chongqing Medical University, Chongqing, China; 4 Institute of Pathology and Southwest Cancer Center, Southwest Hospital, Third Military Medical University (Army Medical University), Chongqing, China

**Keywords:** hepatocellular carcinoma, magnetic resonance imaging, microvascular invasion, prognosis, tertiary lymphoid structures

## Abstract

**Background:**

This study aims to develop a non-invasive MRI-based clinicoradiologic model for predicting microvascular invasion-positive (MVI+) and tertiary lymphoid structures-positive (TLSs+) hepatocellular carcinoma (HCC) and to explore whether this subgroup is associated with different postoperative outcomes and a potential differential association with postoperative TKI-ICI combination therapy.

**Methods:**

A retrospective analysis was conducted on 338 HCC patients across two centers, stratified by MVI and TLSs status, and divided into training (n=237) and validation (n=101) cohorts. MRI features such as tumor size, hemorrhage, and capsule, were assessed. Model performance was assessed using the area under the receiver operating characteristic curve (AUC). Kaplan-Meier curves were used to compare recurrence-free survival (RFS).

**Results:**

The MRI-based clinicoradiologic model for predicting MVI+/TLSs+ HCC (MT-HCC) integrated AFP levels and MRI features, including absence of intratumoral hemorrhage, incomplete capsule, and mosaic structure, achieved an AUC of 0.831 in the validation cohort. In exploratory analyses, the model-predicted MT-HCC group showed a shorter RFS than the model-predicted non-MT-HCC group among patients without postoperative TKI-ICI exposure (p = 0.002), whereas a different RFS pattern was observed among patients with postoperative TKI-ICI exposure (p = 0.017).

**Conclusion:**

The MRI-based clinicoradiologic model may help identify MT-HCC preoperatively and may help identify a subgroup with distinct postoperative prognosis and a possible differential outcome pattern according to postoperative TKI-ICI exposure.

## Introduction

Hepatocellular carcinoma (HCC) is the most common type of primary liver cancer, ranking as the sixth most prevalent cancer and the third leading cause of cancer-related death worldwide (1). For patients with Barcelona Clinic Liver Cancer (BCLC) stage 0 or A, treatment usually involves surgical resection, which offers a 5-year survival rate ranging from 56% to 81%. Nevertheless, even after undergoing radical surgical resection, the recurrence rate remains high at approximately 70% (2). Therefore, reducing post-operative recurrence and improving clinical outcomes are key clinical concerns.

Recent evidence suggests that combining tyrosine kinase inhibitors (TKI) with immune checkpoint inhibitors (ICI) (TKI-ICI combination) shows promise in reducing recurrence and progression in HCC. TKI work by inhibiting pathways such as VEGFR and MAPK, which are involved in tumor angiogenesis and growth, while ICIs restore T-cell function by blocking immune checkpoints like PD-1/PD-L1, enhancing the body’s immune response against tumor cells (3). Clinical trials indicate that TKI-ICI combination therapy may lower recurrence in high-risk patients; nonetheless, approximately 20-30% of patients do not respond to this treatment, with recurrence occurring within 1 year (4, 5). Despite these promising findings, whether postoperative adjuvant TKI-ICI therapy can consistently improve long-term prognosis after radical resection remains insufficiently investigated. Furthermore, identifying appropriate candidates for this treatment—balancing efficacy, cost, and minimizing unnecessary side effects—is an urgent clinical need.

Studies have shown that postoperative TKI offer potential benefits for patients with HCC exhibiting microvascular invasion positive (MVI+) (6–8), likely due to inhibition of MAPK/ERK-mediated proliferation and suppression of VEGF-driven angiogenesis (9). Concurrently, intratumoral tertiary lymphoid structures positive (TLSs+), which resemble secondary lymphoid organs, have emerged as potential biomarkers in cancer immunotherapy, correlating with reduced risk of early HCC relapse and enhanced anti-tumor immunity (10, 11). MVI and TLSs represent two different aspects of HCC biology: vascular dissemination risk and local immune organization. We did not combine them on the assumption that TLSs would counteract the adverse effect of MVI. Rather, we considered the MVI+/TLSs+ phenotype clinically relevant because it reflects the coexistence of high recurrence risk and a pre-existing intratumoral immune architecture. Although this combination does not necessarily imply a more favorable natural course, it may still identify a subgroup with a distinct postoperative outcome pattern, especially in exploratory analyses of postoperative TKI-ICI exposure. Therefore, we used the MVI+/TLSs+ subgroup as a pragmatic target for preoperative prediction and risk stratification, rather than as a definitively established biological subtype.

To our knowledge, no preoperative model has been established to identify MVI+/TLSs+ HCC (MT-HCC). Developing such a model may provide a non-invasive approach for preoperative risk stratification and for exploring whether this subgroup is associated with distinct postoperative outcomes. Therefore, this study aimed (1) to develop an MRI-based clinicoradiologic model to predict MT-HCC, and (2) to explore whether model-predicted subgrouping was associated with distinct postoperative outcomes, including exploratory analyses according to postoperative TKI-ICI combination therapy.

## Patients and methods

### Patient population

**Figure 1:**
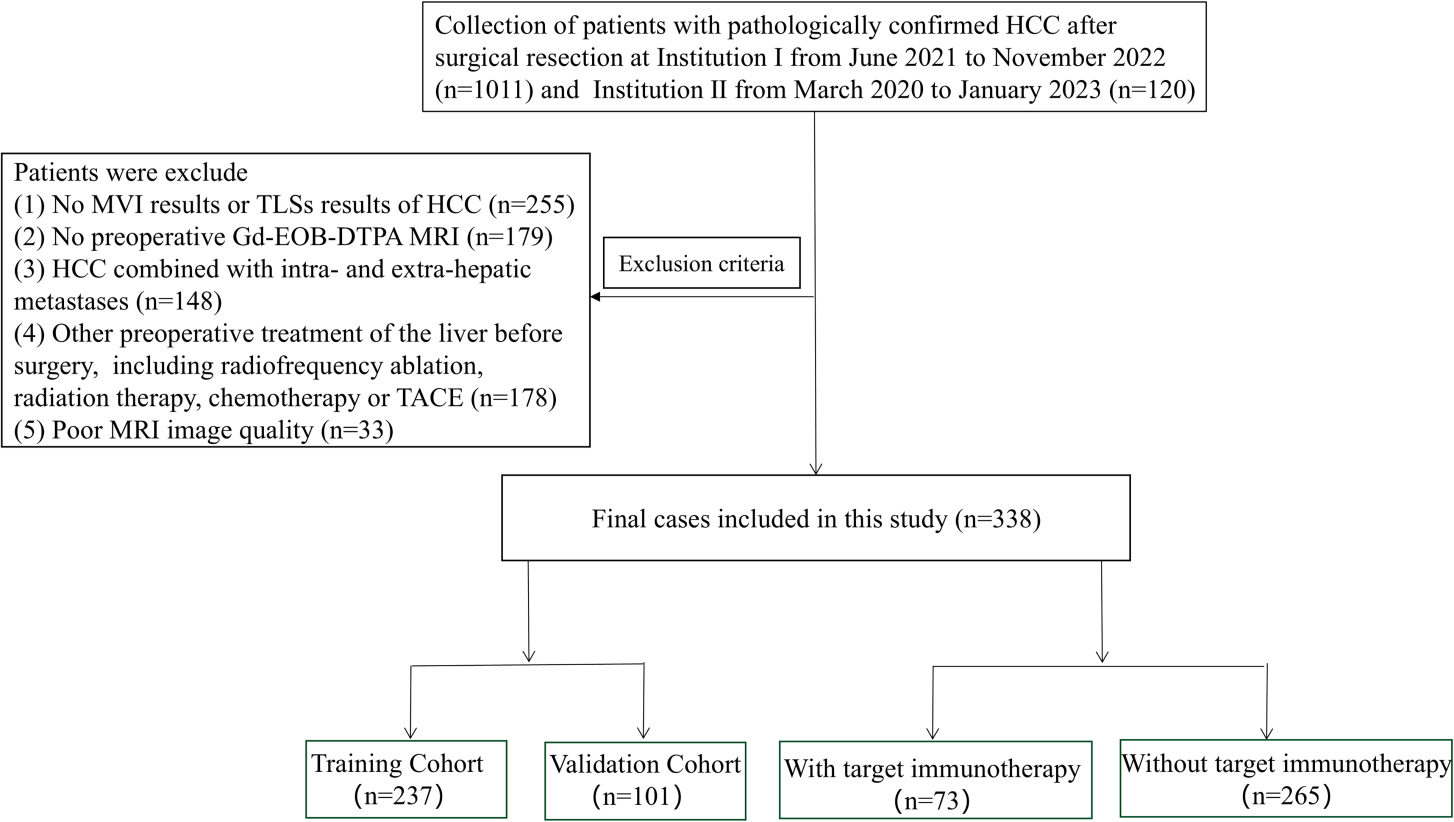
Flow chart of the study population.

A total of 338 consecutive patients with pathologically confirmed HCC after surgical resection were retrospectively enrolled from two tertiary hospitals between March 2020 and January 2023. Inclusion criteria required that each patient had undergone enhanced MRI within one month before surgery, had a surgically resected and pathologically confirmed HCC, and had all necessary clinical and laboratory data available. Exclusion criteria included: (1) any prior history of chemotherapy or loco-regional treatment before surgery; (2) significant imaging artifacts; and (3) with intra- or extra-hepatic metastases. **Figure 1** illustrates the patient selection process diagrammatically.

Because the cohort from the Institution II was relatively small (n = 43), and further subgrouping by MVI/TLS status would have resulted in very small cell counts, we did not use the two institutional cohorts as separate training and validation datasets. Instead, all eligible patients from both institutions were pooled and then randomly assigned in a 7:3 ratio to the training and validation cohorts. The training cohort included 237 patients (122 [51.4%] with MVI+ and 160 (67.5%) with TLSs+; median age of 57 years, interquartile range 50–65 years). The validation cohort comprised 101 patients (45 [44.5%] with MVI+ and 68 [67.3%] with TLSs+; median age of 52 years, interquartile range 47–66 years). Patients were initially classified into four pathological subgroups according to MVI and TLSs status: MVI+/TLSs+, MVI+/TLSs−, MVI−/TLSs+, and MVI−/TLSs−. For model development, the primary binary endpoint was defined as MT-HCC (MVI+/TLSs+) versus non-MT-HCC (the remaining three pathological subgroups).

### Histopathologic data

All surgical specimens from the two institutions were analyzed by two abdominal pathologists (H.R.Z. and Q.R.L., with 22 and 10 years of experience in liver pathology, respectively), who were blinded to all clinical and imaging data. For any discordant cases, a consensus review process was applied. MVI was defined as an endothelium-lined vascular space on microscopy. The presence of TLSs was assessed in at least five hematoxylin and eosin (H&E) sections (range of 5–12 slides per case) which were scanned for whole slide images (WSIs). The degree of TLS maturation in our sections was confirmed by immunochemical staining of CD3, CD20, and CD21. TLSs were classified into three categories based on their stage of maturation: (1) lymphoid aggregates, defined histologically at low-power magnification as tight collections of >100 lymphoid cells with vague, ill-defined clusters (12); (2) primary follicles, which were round clusters of lymphocytes without germinal center formation; and (3) secondary follicles, the most mature form of TLSs with germinal center formation. Any of the above was considered TLSs+. Otherwise, the case was TLSs-.

### Follow-up after surgery

Patients with HCC underwent routine imaging (ultrasound, CT, and/or MRI) every 3–6 months for the first two years after hepatectomy, and then every 6–12 months afterward. Recurrence was determined using standard contrast-enhanced multiphase imaging, characterized by hyperenhancement in the arterial phase and washout in the portal venous or delayed phase (13). Recurrence-free survival (RFS) was defined as the time from surgery to recurrence. The follow-up deadline was May 1, 2024.

### Overview of TKI-ICI combination therapy

TKI-ICI combination therapy was given to 73 HCC patients in the pooled cohort based on previously reported risk factors for a high risk of recurrence after surgery (14). In this study, postoperative TKI-ICI combination therapy was initiated as early as feasible after patients had achieved stable postoperative recovery, had no obvious complications, and had adequate liver function, generally around 4 weeks after surgery. After surgical resection, TKI and ICI treatment were continued for at least 6 months, or until recurrence, data censoring, or discontinuation due to severe adverse events, whichever occurred first. The ICIs used in this cohort mainly included sintilimab (200 mg), tislelizumab (200 mg every 3 weeks), and atezolizumab (1,200 mg every 3 weeks). Sintilimab and tislelizumab are programmed death-1 (PD-1) inhibitors, whereas atezolizumab is a programmed death-ligand 1 (PD-L1) inhibitor. The TKIs mainly included lenvatinib (8 or 12 mg) and donafenib (200 mg). Treatment selection was based on drug availability, reimbursement, local insurance coverage, and patient preference.

Because postoperative TKI-ICI combination therapy was not assigned according to a predefined protocol and regimen selection was heterogeneous, survival analyses involving treatment exposure were considered exploratory.

### MRI protocol

MRI was carried out on two 3.0-T systems (MAGNETOM TrioTim, Siemens, Erlangen, Germany; GE Discovery MR750W, GE Healthcare, Milwaukee, USA). T2-weighted, in-phase and opposed-phase, and T1-weighted 3D liver acquisition with volume acceleration (LAVA) with fat suppression or volume-interpolated body examination (T1-VIBE) were among the sequences. After injecting gadoxetic acid (Primovist, Bayer Schering Pharma, Berlin, Germany) at a rate of 1.0 mL/s and dose of 0.025 mmol/kg, followed by a 30-mL saline chaser. Patients held their breath once the contrast agent reached the aortic arch, allowing for the sequential acquisition of images during the portal venous phase (PVP) and transition phase (55–70 seconds and 150–180 seconds after contrast injection). Susceptibility weighted angiography (SWAN) or susceptibility weighted imaging (SWI) was then carried out. Hepatobiliary phase images were acquired 15 minutes after contrast injection. Detailed scanning parameters are shown in **Supplementary Table 1**.

### Clinical and laboratory data

Picture Archiving and Communications Systems (PACS) were used to obtain clinical and laboratory data on the patients, including age, sex, cause of underlying liver disease, quantification of the hepatitis B virus deoxyribonucleic acid (HBV-DNA), history of antiviral therapy, parameters of liver function and coagulation parameters, alpha-fetoprotein (AFP) level, Barcelona Clinic Liver Cancer (BCLC) stage.

### Imaging data analysis

Three board-certified radiologists (Y.L., X.L., and P.C., with 11, 12, and 31 years of experience in abdominal imaging, respectively, from institution I) independently evaluated the preoperative MRI features, they were blinded to the patient’s clinical information and postoperative pathology results before the assessment. In cases of disagreement, the senior radiologist’s (P.C.) assessment was adopted. The definition of each imaging feature on MRI was documented in **Supplementary Table 2**.

### Statistical analysis

SPSS (IBM SPSS Corp, SPSS Statistics ver. 27.0, USA), MedCalc^®^ Statistical Software version 20.022 (MedCalc Software Ltd, Ostend, Belgium; https://www.medcalc.org; 2021), and R software (version 4.2.2, R Foundation for Statistical Computing, Vienna, Austria) were used for analysis. Inter-reader reliability measures were determined by calculating Fleiss’ kappa values. Inter-reader reliability was assessed using Fleiss kappa values, with values interpreted as follows: 0.00–0.20 indicating slight agreement, 0.21–0.40 fair agreement, 0.41–0.60 moderate agreement, 0.61–0.80 substantial agreement, and 0.81–1.00 almost perfect agreement (15). The Kolmogorov-Smirnov test was used to assess whether the continuous variable data followed a normal distribution. If data followed a normal distribution, they were expressed as mean ± standard deviation, and t-tests were used for comparison between groups, otherwise, they were expressed as median (interquartile range), and Mann–Whitney U tests were used for comparison between groups. Categorical variables were analyzed using the χ^2^ or Fisher's exact tests. Variables associated with MT-HCC were first explored using univariate logistic regression, and those with a p value <0.05 were entered into multivariable logistic regression using the stepwise selection method. Because this approach may yield unstable variable selection and inflated effect estimates, an additional penalized logistic regression analysis (LASSO) was performed as a sensitivity analysis using the same training and validation split. The resulting clinicoradiologic nomogram should therefore be interpreted as exploratory and preliminary. The nomogram’s performance was assessed using the receiver operating characteristic curve (AUC) and 95% confidence interval (CI), with calibration curves and decision curve analysis (DCA) for validation. In addition, exploratory Cox proportional hazards analyses were performed. Among patients who did not receive postoperative TKI-ICI therapy, the four pathological subgroups were compared using the MVI+/TLSs+ subgroup as the reference. In the pooled cohort, an adjusted Cox model including MT-HCC status, postoperative TKI-ICI exposure, and their interaction term was used to explore potential effect modification. Adjustment variables included age, sex, AFP level (> 400 ng/mL), BCLC stage, and tumor size. RFS was analyzed using Kaplan–Meier curves and compared using the log-rank test. A *p*-value <0.05 was considered statistically significant.

## Results

### Clinical characteristics of the training and validation cohorts

Comparison of clinical and pathological characteristics showed no significant differences between the training and validation cohorts except for BCLC stage (**Table 1**).

**Table 1:** Patient clinical and pathologic characteristics of HCC in the training and validation cohorts.

Characteristic	Training cohort, n = 237	Validation cohort, n = 101	*p*
**Sex**			0.796
Male	204 (86.1%)	88 (87.1%)	
Female	33 (13.9%)	13 (12.9%)	
**Age (years)**			0.056
Median (IQR)	57 (50, 65)	52 (47, 66)	
**History of antiviral**			0.175
No	141 (59.5%)	68 (67.3%)	
Yes	96 (40.5%)	33 (32.7%)	
**Etiology**			0.514
HBV	200 (84.4%)	87 (86.1%)	
HCV	6 (2.5%)	1 (1.0%)	
Alcohol	10 (4.2%)	7 (6.9%)	
Other	21 (8.9%)	6 (5.9%)	
**TBIL (μmol/L)**			0.725
Median (IQR)	17 (12, 23)	16 (14, 22)	
**Albumin (g/L)**			0.373
Median (IQR)	39 (33, 42)	39 (35, 43)	
**ALP (U/L)**			0.807
Median (IQR)	84 (67, 108)	84 (68, 104)	
**GGT (U/L)**			0.744
Median (IQR)	49 (30, 86)	46 (29, 78)	
**APTT (s)**			0.684
Median (IQR)	27.50 (26.10, 29.40)	27.40 (25.70, 29.10)	
**PT-INR**			0.302
Median (IQR)	1.03 (0.96, 1.10)	1.00 (0.96, 1.10)	
**PT (s)**			0.503
Median (IQR)	12.00 (11.20, 12.80)	11.80 (11.10, 12.90)	
**FIB (g/L)**			0.163
Median (IQR)	2.50 (2.09, 3.44)	2.39 (1.92, 3.09)	
**TT (s)**			0.171
Median (IQR)	17.80 (16.70, 19.00)	18.20 (16.80, 19.10)	
**HBV-DNA replication≥10^3**			0.711
No	157 (66.2%)	69 (68.3%)	
Yes	80 (33.8%)	32 (31.7%)	
**WBC (x10^9^/L)**			0.665
Median (IQR)	6.6 (4.7, 10.1)	6.8 (4.8, 10.0)	
**PLT (x10^9^/L)**			0.106
Median (IQR)	133 (98, 179)	146 (113, 194)	
**AFP > 400 ng/mL**			0.906
No	184 (77.6%)	79 (78.2%)	
Yes	53 (22.4%)	22 (21.8%)	
**BCLC stage**			0.044
0	31 (13.1%)	19 (18.8%)	
A	155 (65.4%)	71 (70.3%)	
B	23 (9.7%)	2 (2.0%)	
C	28 (11.8%)	9 (8.9%)	
**TLSs**			0.974
Negative	77 (32.5%)	33 (32.7%)	
Positive	160 (67.5%)	68 (67.3%)	
**MVI**			0.244
Negative	115 (48.5%)	56 (55.4%)	
Positive	122 (51.5%)	45 (44.6%)	

*HBV* hepatitis B virus, *HCV* hepatitis C virus, *TBIL* total bilirubin, *ALP* alkaline phosphatase, *GGT* gamma glutamyl transferase, *APTT* activated partial thromboplastin time, *PT-INR* Prothrombin Time International Normalization Ratio, *PT* Prothrombin Time, *FIB* Fibrinogen, *TT* thrombin time, *HBV-DNA* hepatitis B virus-deoxyribonucleic acid, *WBC* white blood cells, *PLT* platelet, *BCLC* Barcelona Clinic Liver Cancer, *AFP* alpha-fetoprotein, *TLSs* tertiary lymphoid structures, *MVI* microvascular invasion. Bold text indicates variable names.

### Clinical characteristics of MT-HCC and non-MT-HCC

The clinical characteristics of MT-HCC and non-MT-HCC are listed in **Table 2**. In the training cohort, except for age, white blood cell count (WBC), AFP and BCLC stage, the clinical characteristics did not differ significantly between MT-HCC and non-MT-HCC groups. Similarly, all clinical characteristics were not significantly different in the validation cohort.

**Table 2:** Patient clinical characteristics of MT-HCC and non-MT-HCC.

Characteristics	Training cohort			Validation cohort		
	Non-MT-HCC, n = 157	MT-HCC, n = 80	*p*	Non-MT-HCC, n = 71	MT-HCC, n = 30	*p*
**Sex**			0.213			0.198
Male	132 (84%)	72 (90%)		64 (90%)	24 (80%)	
Female	25 (16%)	8 (10%)		7 (10%)	6 (20%)	
**Age (years)**			0.017			0.378
Mean ± SD	58 ± 9	54 ± 12		55 ± 12	53 ± 11	
**History of antiviral**			0.655			0.710
Yes	62 (39%)	34 (43%)		24 (34%)	9 (30%)	
No	95 (61%)	46 (58%)		47 (66%)	21 (70%)	
**Etiology**			0.069			0.138
HBV	131 (83%)	69 (86%)		61 (86%)	26 (87%)	
HCV	3 (2%)	3 (4%)		0 (0%)	1 (3%)	
Alcohol	10 (6%)	0 (0%)		4 (6%)	3 (10%)	
Other	13 (8%)	8 (10%)		6 (8%)	0 (0%)	
**TBIL (μmol/L)**			0.607			0.494
Median (IQR)	17 (12, 23)	18 (13, 22)		16 (14, 24)	16 (13, 20)	
**Albumin (g/L)**			0.065			0.568
Mean ± SD	39 ± 6	37 ± 6		39 ± 5	38 ± 9	
**ALP (U/L)**			0.939			0.314
Median (IQR)	84 (69, 106)	86 (63, 117)		88 (69, 105)	82 (67, 95)	
**GGT (U/L)**			0.398			0.314
Median (IQR)	48 (29, 81)	55 (33, 91)		46 (29, 77)	55 (38, 77)	
**APTT (s)**			0.364			0.861
Mean ± SD	27.8 ± 3.2	28.3 ± 3.6		27.92 ± 3.04	27.81 ± 2.83	
**PT-INR**			0.330			0.473
Mean ± SD	1.04 ± 0.11	1.06 ± 0.11		1.03 ± 0.12	1.05 ± 0.14	
**PT (s)**			0.246			0.917
Median (IQR)	12.00 (11.20,12.80)	12.00 (11.40,13.00)		12.00 (11.15,12.85)	11.75 (11.00,12.93)	
**FIB (g/L)**			0.430			0.944
Mean ± SD	2.89 ± 1.07	3.02 ± 1.34		2.88 ± 1.43	2.91 ± 2.07	
**TT (s)**			0.655			0.916
Mean ± SD	17.84 ± 1.91	17.74 ± 1.50		18.17 ± 2.24	18.13 ± 1.20	
**PTINR/Alb**			0.967			0.560
Median (IQR)	0.03 (0.02, 0.04)	0.03 (0.02, 0.04)		0.03 (0.02, 0.04)	0.03 (0.02, 0.04)	
**HBV-DNA** **replication≥10^3^**			0.383			0.813
No	101 (64%)	56 (70%)		48 (68%)	21 (70%)	
Yes	56 (36%)	24 (30%)		23 (32%)	9 (30%)	
**WBC (x10^9^/L)**			0.020			0.225
Mean ± SD	7.4 ± 3.9	8.9 ± 4.7		7.8 ± 4.8	9.1 ± 4.9	
**PLT (x10^9^/L)**			0.314			0.973
Mean ± SD	140 ± 60	149 ± 65		153 ± 54	152 ± 56	
**AFP > 400 ng/mL**			0.003			0.439
No	131 (83%)	53 (66%)		57 (80%)	22 (73%)	
Yes	26 (17%)	27 (34%)		14 (20%)	8 (27%)	
**BCLC stage**			0.005			0.314
0	23 (15%)	8 (10%)		14 (20%)	5 (17%)	
A	104 (66%)	51 (64%)		51 (72%)	20 (67%)	
B	19 (12%)	4 (5%)		2 (3%)	0 (0%)	
C	11 (7%)	17 (21%)		4 (6%)	5 (17%)	

*HBV* hepatitis B virus, *HCV* hepatitis C virus, *TBIL* total bilirubin, *ALP* alkaline phosphatase, *GGT* gamma glutamyl transferase, *APTT* activated partial thromboplastin time, *PT-INR* Prothrombin Time International Normalization Ratio, *PT* Prothrombin Time, *FIB* Fibrinogen, *TT* thrombin time, *HBV-DNA* hepatitis B virus-deoxyribonucleic acid, *WBC* white blood cells, *PLT* platelet, *AFP* alpha-fetoprotein, *BCLC* Barcelona Clinic Liver Cancer. Bold text indicates variable names.

### Association between MRI features and MT-HCC

Inter-rater reliability between the three radiologists demonstrated substantial to perfect agreement (Fleiss’κ = 0.602–1.000), as shown in **Supplementary Table 3**. Gadoxetic acid-enhanced MRI features in the training cohort and validation cohorts were summarized in **Table 3**. In the training cohort, MRI features such as regular shape, intratumoral hemorrhage, and complete capsule were observed significantly more often in the non-MT-HCC group than in the MT-HCC group. In contrast, MT-HCC showed a significantly higher frequency of arterial phase peritumoral enhancement, intratumoral necrosis or ischemia (>20%), tumor in vein, and mosaic structure compared to the non-MT-HCC group.

**Table 3:** The gadoxetic acid-enhanced MRI features of MT-HCC and non-MT-HCC.

Characteristics	Training cohort			Validation cohort		
	Non-MT-HCC, n = 157	MT-HCC, n = 80	p	Non-MT-HCC, n = 71	MT-HCC, n = 30	p
**Size (cm)**			0.288			0.893
Mean ± SD	3.94 ± 2.15	4.24 ± 2.00		4.00 ± 2.14	3.94 ± 1.94	
**Regular shape**			0.010			0.002
Yes	35 (22%)	7 (9%)		26 (37%)	2 (7%)	
No	122 (78%)	73 (91%)		45 (63%)	28 (93%)	
**Intratumor fat**			0.142			0.381
Absence	135 (86%)	74 (93%)		58 (82%)	27 (90%)	
Presence	22 (14%)	6 (8%)		13 (18%)	3 (10%)	
**Intratumor hemorrhage**			0.010			0.033
Absence	91 (58%)	60 (75%)		41 (58%)	24 (80%)	
Presence	66 (42%)	20 (25%)		30 (42%)	6 (20%)	
**Arterial phase peritumoral enhancement**			<0.001			0.013
Absence	129 (82%)	47 (59%)		59 (83%)	18 (60%)	
Presence	28 (18%)	33 (41%)		12 (17%)	12 (40%)	
**Complete capsule**			<0.001			<0.001
Absence	74 (47%)	69 (86%)		26 (37%)	26 (87%)	
Presence	83 (53%)	11 (14%)		45 (63%)	4 (13%)	
**Intratumor necrosis** **or ischemia (>20%)**			0.017			0.010
Absence	116 (74%)	47 (59%)		54 (76%)	15 (50%)	
Presence	41 (26%)	33 (41%)		17 (24%)	15 (50%)	
**Intratumor necrosis** **or ischemia (>50%)**			0.061			0.001
Absence	135 (86%)	61 (76%)		64 (90%)	19 (63%)	
Presence	22 (14%)	19 (24%)		7 (10%)	11 (37%)	
**Satellite nodule**			0.582			>0.999
Absence	129 (82%)	68 (85%)		65 (92%)	27 (90%)	
Presence	28 (18%)	12 (15%)		6 (8%)	3 (10%)	
**Peritumoral** **HBP hypointensity**			0.132			0.010
Absence	104 (66%)	45 (56%)		50 (70%)	13 (43%)	
Presence	53 (34%)	35 (44%)		21 (30%)	17 (57%)	
**Tumor in vein**			<0.001			0.007
Absence	139 (89%)	56 (70%)		68 (96%)	23 (77%)	
Presence	18 (11%)	24 (30%)		3 (4%)	7 (23%)	
**Rim APHE**			0.120			<0.001
Absence	131 (83%)	60 (75%)		65 (92%)	19 (63%)	
Presence	26 (17%)	20 (25%)		6 (8%)	11 (37%)	
**Mosaic structure**			0.018			0.159
Absence	68 (43%)	22 (28%)		32 (45%)	9 (30%)	
Presence	89 (57%)	58 (73%)		39 (55%)	21 (70%)	
**Intratumor artery**			0.371			0.613
Absence	111 (71%)	52 (65%)		46 (65%)	21 (70%)	
Presence	46 (29%)	28 (35%)		25 (35%)	9 (30%)	

*MT* microvascular invasion positive and tertiary lymphoid structures positive, *HCC* hepatocellular carcinoma, *HBP* hepatobiliary phase, *APHE* arterial phase hyperenhancement. Bold text indicates variable names.

After parameter selection, AFP > 400 ng/mL (OR, 1.84), absence of intratumoral hemorrhage (OR, 4.86), incomplete capsule (OR, 9.43) and mosaic structure (OR, 2.49) were confirmed as independent significant variables associated with MT-HCC.

### Development and validation of the diagnostic model

**Figure 2:**
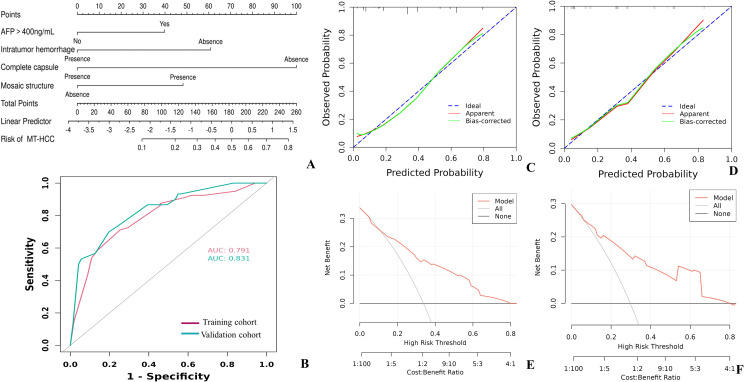
Nomogram for predicting MVI+/TLSs+ HCC (MT-HCC) risk, and evaluation of the nomogram model in the training cohort and validation cohort. **(A)** A point scale from 0 to 100 was used to score each variable, then the sum of the scores was calculated. The risk of MT-HCC could be predicted by looking at the total points axis. **(B)** The model demonstrated high predictive accuracy and discrimination, with an AUC of 0.791 in the training cohort and an AUC of 0.831 in the validation cohort. The calibration curve for the nomogram indicated good consistency between the actual and the predicted probability in the training cohort **(C)** and validation cohort **(D)**. **(E, F)** The DCA suggested potential net benefit of the model within the present dataset. MVI = microvascular invasion, TLSs, tertiary lymphoid structures; HCC, hepatocellular carcinoma; AUC, area under the receiver operating characteristic curve; DCA, decision curve analysis.

A clinicoradiologic nomogram was constructed based on the significant variables from multivariable analyses, including AFP > 400 ng/mL, absence of intratumoral hemorrhage, incomplete capsule, and mosaic structure. In the training cohort, the diagnostic model achieved an AUC of 0.791 (95% CI: 0.729–0.853), with sensitivity, specificity, and accuracy of 71.3%, 74.5%, and 73.4%, respectively. In the validation cohort, the AUC was 0.831 (95% CI: 0.735–0.928), with sensitivity, specificity, and accuracy of 70.0%, 80.3%, and 77.2%, respectively. Calibration plots of the nomogram showed good agreement between estimated and actual observations in both training and validation cohorts. DCA for the nomogram indicates that the nomogram improves the benefit compared with the measures that treat all patients and treat no patient when threshold probability is 0.16~0.8 in the training cohort, and 0.05~0.8 in the internal validation cohort, the DCA suggested potential net benefit across a range of threshold probabilities in the training cohort and the internal validation cohort (**Figure 2**).

Because BCLC stage differed significantly between the training and validation cohorts, an additional sensitivity analysis was performed in the validation cohort after adjusting for BCLC stage. The original clinicoradiologic model showed an AUC of 0.831 (95% CI, 0.735–0.928). After incorporating BCLC stage into the validation analysis, the AUC was 0.826 (95% CI, 0.728–0.924). These findings suggest that the imbalance in BCLC stage distribution did not materially affect the discriminative performance of the model in the validation cohort. Detailed results are provided in **Supplementary Table 4**.

As a sensitivity analysis, we additionally performed a penalized logistic regression analysis using LASSO with the same training and validation split. The LASSO-based model retained complete capsule, intratumoral hemorrhage, age, WBC, and PT as predictors. In the training cohort, the LASSO model achieved an AUC of 0.792 (95% CI, 0.727–0.857), with sensitivity, specificity, and accuracy of 83.8%, 62.4%, and 69.6%, respectively. In the validation cohort, the AUC was 0.840 (95% CI, 0.745–0.935), with sensitivity, specificity, and accuracy of 83.3%, 70.4%, and 74.3%, respectively. These findings support consistency in predictive performance rather than stability of variable selection, because the retained variables were not completely identical to those in the original stepwise model. Detailed results are provided in **Supplementary Table 5**.

### Associations of pathological/model-defined subgrouping with postoperative outcomes

Of the 338 patients in the pooled cohort, 73 patients received TKI-ICI combination therapy postoperatively, the estimated 1-year, 2-year and 3-year RFS rates after TKI-ICI combination therapy for HCC were 90.1%, 63.8% and 51.1%, respectively. 265 patients did not receive TKI-ICI combination therapy postoperatively, the estimated 1-year, 2-year and 3-year RFS rates for HCC were 72.8%, 48.9% and 35.0%, respectively.

**Figure 3:**
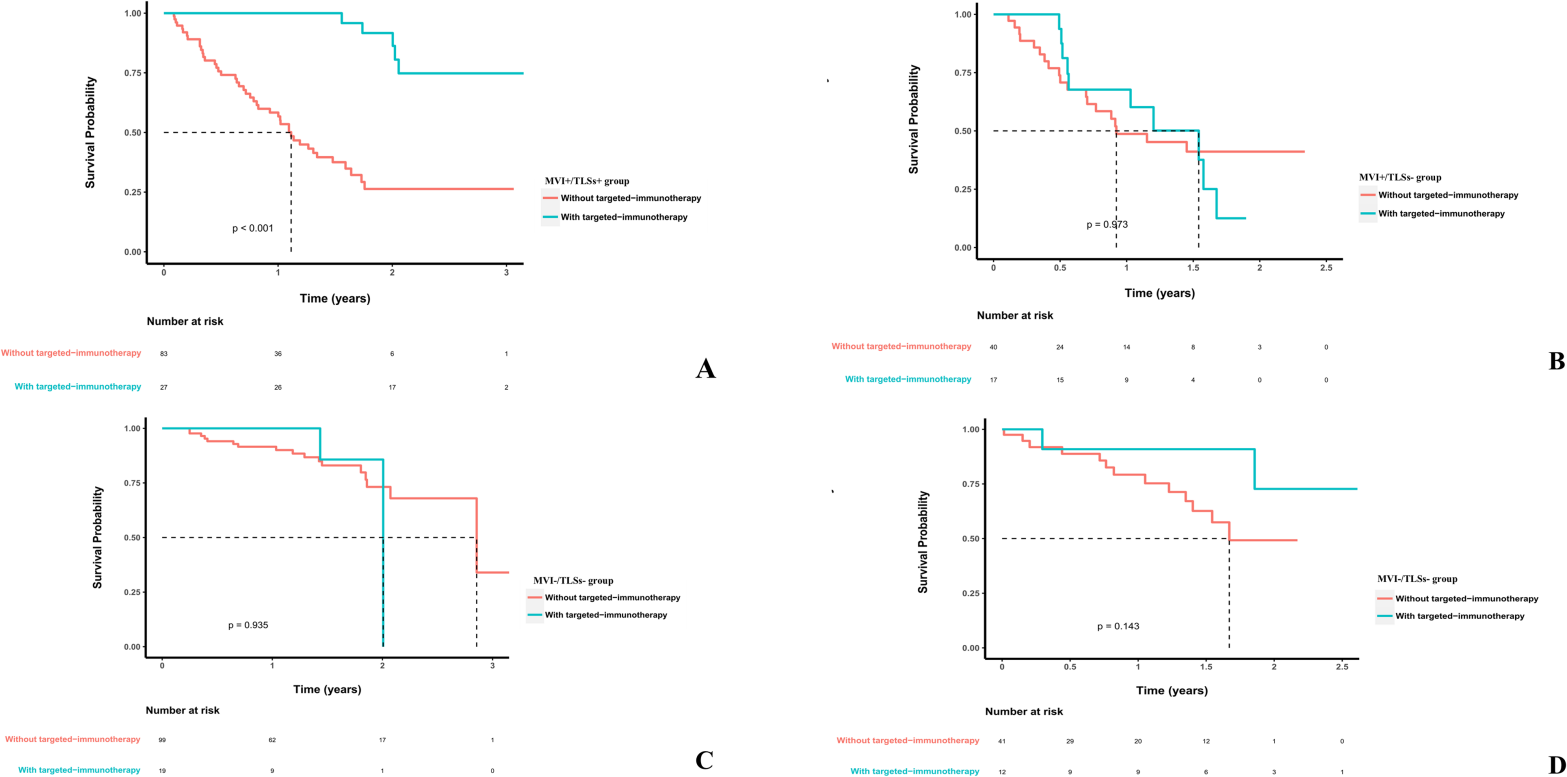
The survival prognosis of the intra-subgroups (MVI+/TLSs+, MVI+/TLSs-, MVI-/TLSs+, MVI-/TLSs-) with and without TKI-ICI combination therapy. Except for **(A)** MVI+/TLSs+ group, none of the other groups, including the **(B)** MVI+/TLSs-, **(C)** MVI-/TLSs+, and **(D)** MVI-/TLSs- groups showed a significant RFS difference according to postoperative TKI-ICI combination therapy. MVI, microvascular invasion; TLS, tertiary lymphoid structures; HCC, hepatocellular carcinoma; TKI, tyrosine kinase inhibitors; ICI, immune checkpoint inhibitors.

**Figure 4:**
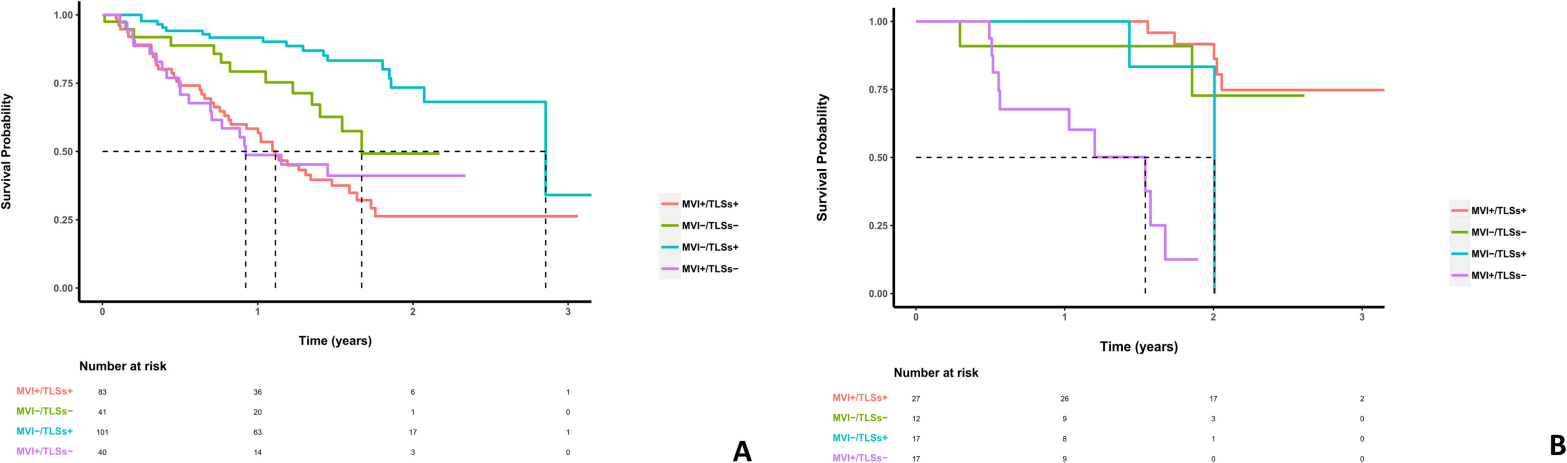
The RFS of the inter-subgroups (MVI+/TLSs+, MVI+/TLSs-, MVI-/TLSs+, MVI-/TLSs-) with or without TKI-ICI combination therapy. **(A)** The RFS of the MVI+/TLSs+ group without TKI-ICI combination therapy was lower than the MVI-/TLSs+ group and the MVI-/TLSs- group (*p* = 0.038, *p* < 0.001), but not significantly different from the MVI+/TLSs- group (*p* = 0.769). **(B)** In patients receiving postoperative TKI-ICI combination therapy, the MVI+/TLSs+ group showed a more favorable RFS pattern, particularly compared with the MVI+/TLSs− group. MVI, microvascular invasion; TLS, tertiary lymphoid structures; TKI, tyrosine kinase inhibitors; ICI, immune checkpoint inhibitors; RFS, recurrence-free survival.

Within the MVI+/TLSs+ subgroup, postoperative TKI-ICI combination therapy was associated with longer RFS than no postoperative TKI-ICI combination therapy (*p* < 0.001; **Figure 3A**). This association was not observed in the other pathological subgroups (**Figures 3B–D**). We then compared RFS across the four pathological subgroups separately according to postoperative TKI-ICI exposure. Among patients who did not receive postoperative TKI-ICI combination therapy, the MVI+/TLSs+ group showed shorter RFS than the MVI−/TLSs+ group and the MVI−/TLSs− group (*p* = 0.038 and *p* < 0.001, respectively), whereas no significant difference was observed compared with the MVI+/TLSs− group (*p* = 0.769) (**Figure 4A**). Among patients who received postoperative TKI-ICI combination therapy, RFS in the MVI+/TLSs+ group was not significantly different from that in the MVI−/TLSs+ group or the MVI−/TLSs− group (*p* = 0.201 and *p* = 0.518, respectively), but was significantly longer than that in the MVI+/TLSs− group (*p* < 0.001) (**Figure 4B**).

In the subgroup of patients who did not receive postoperative TKI-ICI therapy, exploratory Cox regression showed no significant RFS difference between the MVI+/TLSs+ and MVI+/TLSs− groups (adjusted HR, 1.01; 95% CI, 0.57–1.79; *p* = 0.971), whereas the MVI−/TLSs+ group had a significantly lower recurrence risk than the MVI+/TLSs+ group (adjusted HR, 0.28; 95% CI, 0.16–0.51; *p* < 0.001). The difference between the MVI−/TLSs− and MVI+/TLSs+ groups was not statistically significant after adjustment (adjusted HR, 0.80; 95% CI, 0.41–1.56; *p* = 0.512). In the pooled cohort, an adjusted Cox model including MT-HCC status, postoperative TKI-ICI exposure, and their interaction term showed significant effect modification between MT-HCC and treatment exposure (interaction HR, 0.12; 95% CI, 0.04–0.38; *p* = 0.0003). In stratified adjusted analyses, postoperative TKI-ICI exposure was associated with lower recurrence risk in the MT-HCC subgroup (adjusted HR, 0.13; 95% CI, 0.05–0.34; *p* < 0.001), but not in the non-MT-HCC subgroup (adjusted HR, 1.08; 95% CI, 0.57–2.02; *p* = 0.822). Detailed results are provided in **Supplementary Tables 6**–**8**.

**Figure 5:**
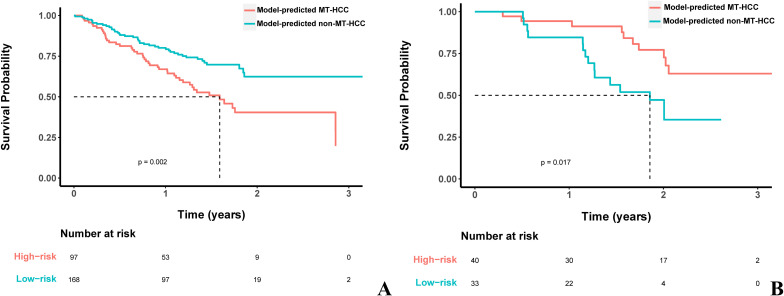
Kaplan–Meier analysis of RFS based on the MRI-based clinicoradiologic model. **(A)** Among patients who did not receive postoperative TKI-ICI combination therapy, the model-predicted MT-HCC group had significantly shorter RFS than the model-predicted non-MT-HCC group (p = 0.002). **(B)** Among patients who received postoperative TKI-ICI combination therapy, the model-predicted MT-HCC group showed a more favorable RFS pattern than the model-predicted non-MT-HCC group (p = 0.017). MT-HCC, MVI+/TLSs+ HCC; TKI, tyrosine kinase inhibitors; ICI, immune checkpoint inhibitors; RFS, recurrence-free survival.

The pooled cohort was further stratified according to the MRI-based clinicoradiologic model. Patients were classified as model-predicted MT-HCC or model-predicted non-MT-HCC based on the optimal cut-off value of the nomogram. Among patients who did not receive TKI-ICI combination therapy, the model-predicted MT-HCC group had significantly shorter RFS than the model-predicted non-MT-HCC group (*p* = 0.002) (**Figure 5A**). In contrast, among patients who received postoperative TKI-ICI combination therapy, the model-predicted MT-HCC group showed a more favorable RFS pattern than the model-predicted non-MT-HCC group (*p* = 0.017) (**Figure 5B**).

## Discussion

In this study, we investigated the association of preoperative clinicoradiologic variables with MT-HCC and developed an MRI-based clinicoradiologic model to predict MT-HCC preoperatively. The results revealed that AFP level (> 400 ng/mL) and several MRI features, including absence of intratumoral hemorrhage, incomplete capsule, and mosaic structure, were significantly associated with MT-HCC. The nomogram constructed from these variables demonstrated good discriminative ability, with AUC values of 0.791 in the training cohort and 0.831 in the validation cohort. The observed sensitivity and specificity suggest potential utility for preoperative risk stratification. In addition, the penalized logistic regression sensitivity analysis showed comparable discrimination in the validation cohort. However, because the retained variables differed from those in the original stepwise model, this finding supports consistency in predictive performance rather than stability of variable selection. Therefore, the present model should still be interpreted as exploratory and preliminary. Our MRI-based clinicoradiologic model may provide a non-invasive approach to exploratory risk stratification in HCC, particularly for identifying patients with MT-HCC, a subgroup associated with distinct postoperative prognosis and a potentially different RFS pattern in exploratory analyses involving postoperative TKI-ICI exposure.

HCC with MVI+ has a greater propensity for vascular dissemination and higher postoperative recurrence risk (16), whereas TLSs are generally linked to antitumor immune activity (17). In our cohort, however, the MVI+/TLSs+ and MVI+/TLSs− groups showed no significant difference in RFS in patients who did not receive postoperative TKI-ICI combination therapy. This finding suggests that the favorable prognostic effect usually attributed to TLSs may be weakened in the setting of MVI-positive tumor biology. A possible explanation is that MVI-positive tumors may exhibit a more proliferative and immunosuppressive microenvironment, which could limit the protective significance of TLSs in the natural postoperative course (18). Consistent with this possibility, immunoregulatory stromal and immune cell populations, including TREM2+ macrophages and cycling T cells, have been reported to be enriched in the MVI-positive microenvironment (16). Therefore, in the present study, the MVI+/TLSs+ subgroup should be interpreted as a pragmatic and exploratory framework for postoperative risk stratification and treatment-related analysis, rather than as a definitive biological subtype. Our additional exploratory analyses further support this interpretation. The rationale for the MT-HCC endpoint does not lie in a simple additive prognostic effect of MVI and TLSs, but rather in the distinct risk structure and treatment-related outcome pattern associated with their coexistence.

Imaging features, including incomplete capsule and mosaic structure, are important for predicting more aggressive tumor phenotypes. The capsule generally served as a barrier to tumor invasion, an incomplete capsule suggested that the tumor has already breached this barrier, and protruded into the peritumoral non-neoplastic parenchyma (19). Mosaic architecture was usually observed in large tumors and was associated with tumor heterogeneity. Greater heterogeneity within larger tumors was typically associated with regions of necrosis, fatty metamorphosis, and different cellular populations (20). Hemorrhage was more frequent in large, progressed HCCs as a result of vascular invasion and rupture. The imaging feature of intratumoral hemorrhage has been identified as an independent risk factor for predicting MVI+ and TLSs- status (21–23), which was in contradiction with the results of our study. Whether this discrepancy was related to the interaction between intratumoral vasculature and the immune microenvironment warranted further investigation. High AFP level was associated with aggressive tumor behavior and a poor prognosis, which was in accordance with previous studies (24, 25), our study confirmed that a high serum AFP level (>400 ng/mL) was an independent predictor of MT-HCC.

The timing of the postoperative TKI-ICI combination therapy is a clinically relevant issue, given the possibility of metastatic spread to other liver segments after anatomical hepatectomy (26), or residual tumor foci after non-anatomical hepatectomy (27, 28). In the present study, exploratory analyses of the pooled cohort showed that, across pathological subgrouping and model-based stratification, a more favorable RFS pattern associated with postoperative TKI-ICI combination therapy was observed mainly in the MT-HCC subgroup. To our knowledge, this specific association has not been clearly described previously. A possible biological explanation is that TKI and ICI may exert complementary effects in this setting. Anti-vascular endothelial growth factor-targeted therapies can modulate the tumor microenvironment, including effects on hypoxia and immune suppression (29), whereas TLSs may reflect a more organized local immune contexture characterized by enhanced immune-cell infiltration, antigen presentation, and lymphocyte activation (30). Under this framework, MT-HCC may represent a clinically relevant subgroup for postoperative risk stratification and exploratory treatment-related assessment. However, these findings should be interpreted cautiously and regarded as hypothesis-generating rather than definitive evidence for treatment selection.

This study has several limitations. First, this was a retrospective multicenter study, and selection bias could not be completely avoided. In particular, the treatment-related analyses were non-randomized, treatment allocation was influenced by clinical risk assessment and non-protocol factors, and confounding by indication cannot be excluded. In addition, no propensity score matching or inverse probability weighting was performed, and therefore residual imbalance between treated and untreated patients may have remained. Second, MRI features were assessed visually, which may introduce subjectivity despite substantial to almost perfect inter-reader agreement. Future studies using radiomics or deep learning may improve objectivity and reproducibility. Third, postoperative TKI-ICI regimens were heterogeneous, and some subgroup-based survival analyses involved relatively small sample sizes. Finally, although patients were enrolled from two institutions, model evaluation was based on a random split of the pooled dataset and therefore constituted internal validation rather than true independent external validation. As a result, the generalizability and clinical applicability of the model remain uncertain. Larger prospective multicenter studies with genuine external validation are needed before clinical implementation.

In conclusion, our MRI-based clinicoradiologic model showed promising performance for preoperative identification of MT-HCC. The model may assist risk stratification and may help identify a subgroup with distinct postoperative prognosis and a possible differential outcome pattern according to postoperative TKI-ICI exposure. Because treatment-related analyses were observational and exploratory, these findings should be interpreted as hypothesis-generating and require confirmation in larger prospective protocol-based studies before clinical implementation.

## Data Availability

The data analyzed in this study is subject to the following licenses/restrictions: The datasets used and analysed during the current study are available from the corresponding author on reasonable request. Requests to access these datasets should be directed to lxm359261069@tmmu.edu.cn.
