# Supplementary material for: MRI-based clinicoradiologic model for identifying MT-HCC with distinct postoperative prognosis and potential association with postoperative TKI-ICI therapy
Source: Front Immunol. 2026 May 8;17:1806601. doi: 10.3389/fimmu.2026.1806601 (PMC13194456; doi:10.3389/fimmu.2026.1806601)
Supplement: Supplementary file 1 [file Table1.docx]

**Supplementary**

**Table S1** MRI sequence parameters in the training and external validation cohorts

| **Sequence** | **Plane** | **TR** | **TE** | **FA** | **FOV (mm)** | **Slice (mm)** | **Matrix** |
| --- | --- | --- | --- | --- | --- | --- | --- |
| **Training cohort** |  |  |  |  |  |  |  |
| T2-HASTE | Axial | 700 | 90 | 100 | 340 | 8 | 320x195 |
| In-opp-GRE | Axial | 206 | 2.54/3.83 | 65 | 387 | 7 | 256x131 |
| T1-3D WIBE  (pre-contrast; dynamic phases; HBP) | Axial | 3 | 1 | 13 | 400 | 2.5 | 320x154 |
| SWI | Axial | 150 | 10 | 20 | 380 | 5 | 384x187 |
| **External validation cohort** |  |  |  |  |  |  |  |
| T2-SSFSE | Axial | 4000 | 97.7 | 142 | 380 | 5 | 320x320 |
| In-opp-GRE | Axial | 225 | 2.5/1.2 | 60 | 380 | 6 | 288x192 |
| T1-LAVA  (pre-contrast; dynamic phases; HBP) | Axial | 4.3 | 1.8 | 10 | 400 | 4 | 256x192 |
| SWAN | Axial | minimum | out of phase | 15 | 240 | 6 | 380x380 |

TR repetition time, TE echo time, FA flip angle, FOV field of view, Slice slice thickness, HASTE half-fourier acquisition single-shot fast spin echo sequence, FGRE fast gradient echo, VIBE volume interpolated body examination, HBP hepatobiliary phases, SWI susceptibility weighted imaging, SWAN susceptibility weighted angiography.

**Table S2** Definition of features and representative gadoxetic acid-enhanced MRI

| Imaging features | Definition |
| --- | --- |
| Tumor size | The maximum diameter of the lesion in axial imaging |
| Irregular shape | Irregular tumor margin protruding into the liver parenchyma |
| Intratumor fat | Loss of signal in out-phase |
| Intratumor hemorrhage | High signal on T1-weighted images in the tumor and low signal on SWI/SWAN sequence |
| Arterial phase peritumoral enhancement | Early arterial phase wedge-shaped or irregular and circumferential enhancement in parenchyma adjacent to the tumor that fades during later phase |
| Rim APHE | An irregular enhancement outside the tumor margin with broad contact with the tumor border in the arterial phase, and becoming isointensity with background liver parenchyma in the delayed phase |
| Intratumor necrosis or ischemia | High signal intensity on T2-weighted images without enhancement on postcontrast T1-weighted images and involving at least 20% or 50% of the tumor area at the level of the largest cross-sectional diameter |
| Complete capsule | Peripheral rim of smooth hyperenhancement in the portal venous or delay phase |
| Peritumoral HBP hypointensity | Wedge-shaped or flame-like hypointensity area of hepatic parenchyma outside of the tumor margin on HBP |
| Satellite nodule | single or multiple nodules < 1 cm in diameter and within 2 cm of the main lesion |
| Tumor in vein | Unequivocal enhancing soft tissue in the portal vein or its branches |
| Mosaic architecture | Presence of randomly distributed internal nodules or compartments, usually with different imaging features |
| Intratumor artery | Discrete arteries within the tumor on the arterial phase images |

SWI susceptibility weighted imaging, SWAN susceptibility weighted angiography, APHE arterial phase hyperenhancement, HBP hepatobiliary phase.

**Table S3** Interobserver agreement of gadoxetic acid-enhanced MRI features of HCC

| Gadoxetic acid-enhanced MRI features | Fleiss κ |
| --- | --- |
| Tumor size | 1.000 |
| Regular shape | 0.803 |
| Intratumor fat | 0.836 |
| Intratumor hemorrhage | 0.730 |
| Arterial phase peritumoral enhancement | 0.665 |
| Complete capsule | 0.673 |
| Intratumor necrosis or ischemia (>20%) | 0.626 |
| Intratumor necrosis or ischemia (>50%) | 0.763 |
| Satellite nodule | 0.614 |
| Peritumor HBP hypointensity | 0.657 |
| Tumor in vein | 0.679 |
| Rim APHE | 0.742 |
| Mosaic structure | 0.670 |
| Intratumor artery | 0.602 |

**Table S4.** Sensitivity analysis of validation-cohort model performance after incorporating BCLC stage

| **Model evaluated in the validation cohort** | **AUC** | **95% CI** | **Sensitivity (%)** | **Specificity (%)** | **Accuracy (%)** |
| --- | --- | --- | --- | --- | --- |
| Original clinicoradiologic model | 0.831 | 0.735–0.928 | 70.0 | 80.3 | 77.2 |
| Original clinicoradiologic model+BCLC stage | 0.826 | 0.728–0.924 | 80.0 | 73.2 | 75.2 |

**Abbreviations:** AUC, area under the receiver operating characteristic curve; CI, confidence interval; BCLC, Barcelona Clinic Liver Cancer.

**Table S5. Penalized logistic regression (LASSO) sensitivity analysis for prediction of MT-HCC**

| **Item** | **Training cohort** | **Validation cohort** |
| --- | --- | --- |
| AUC (95% CI) | 0.792 (95% CI, 0.727–0.857) | 0.840 (95% CI, 0.745–0.935) |
| Sensitivity (%) | 83.8 | 83.3 |
| Specificity (%) | 62.4 | 70.4 |
| Accuracy (%) | 69.6 | 74.3 |

**Selected predictors retained by LASSO:** complete capsule, intratumoral hemorrhage, age, WBC, and PT.

**Table S6.** Exploratory Cox regression analyses of recurrence-free survival among the four pathological subgroups in patients without postoperative TKI-ICI therapy

| **Pathological subgroup** | **No. of patients** | **No. of recurrences** | **Unadjusted HR (95% CI)** | ***p*** | **Adjusted HR (95% CI)** | ***p*** |
| --- | --- | --- | --- | --- | --- | --- |
| MVI+/TLSs+ | 83 | 44 | Reference | – | Reference | – |
| MVI+/TLSs− | 40 | 19 | 1.05 (0.61–1.80) | 0.859 | 1.01 (0.57–1.79) | 0.971 |
| MVI−/TLSs+ | 101 | 17 | 0.22 (0.12–0.38) | <0.001 | 0.28 (0.16–0.51) | <0.001 |
| MVI−/TLSs− | 41 | 13 | 0.59 (0.32–1.10) | 0.097 | 0.80 (0.41–1.56) | 0.512 |

**Abbreviations:** HR, hazard ratio; CI, confidence interval; MVI, microvascular invasion; TLSs, tertiary lymphoid structures; TKI, tyrosine kinase inhibitor; ICI, immune checkpoint inhibitor.

**Note:** The MVI+/TLSs+ subgroup was used as the reference category. Adjusted analyses were adjusted for age, sex, AFP level (>400 ng/mL), BCLC stage, and tumor size.

**Table S7.** Adjusted Cox model for recurrence-free survival in the pooled cohort including MT-HCC status, postoperative TKI-ICI exposure, and their interaction

| **Variable** | **Adjusted HR** | **95% CI** | ***p*** |
| --- | --- | --- | --- |
| MT-HCC | 2.00 | 1.30–3.07 | 0.0016 |
| Postoperative TKI-ICI exposure | 1.03 | 0.55–1.90 | 0.934 |
| MT-HCC × postoperative TKI-ICI exposure | 0.12 | 0.04–0.38 | 0.0003 |
| Age | 1.00 | 0.98–1.01 | 0.579 |
| Female sex | 0.77 | 0.40–1.51 | 0.454 |
| AFP >400 ng/mL | 1.23 | 0.79–1.90 | 0.356 |
| Tumor size (per cm) | 1.24 | 1.12–1.37 | <0.001 |
| BCLC stage 0 vs A | 1.17 | 0.54–2.52 | 0.691 |
| BCLC stage B vs A | 1.40 | 0.73–2.69 | 0.307 |
| BCLC stage C vs A | 2.67 | 1.60–4.47 | <0.001 |

**Abbreviations:** HR, hazard ratio; CI, confidence interval; MT-HCC, MVI+/TLSs+ hepatocellular carcinoma; TKI, tyrosine kinase inhibitor; ICI, immune checkpoint inhibitor; AFP, alpha-fetoprotein; BCLC, Barcelona Clinic Liver Cancer.

**Note:** The model included MT-HCC status, postoperative TKI-ICI exposure, and their interaction term. Reference categories were non-MT-HCC, no postoperative TKI-ICI exposure, male sex, AFP ≤400 ng/mL, and BCLC stage A. The model was adjusted for age, sex, AFP level (>400 ng/mL), BCLC stage, and tumor size.

**Table S8.** Stratified adjusted Cox analyses of postoperative TKI-ICI exposure according to MT-HCC status

| **Subgroup** | **No. of patients** | **No. of recurrences** | **Adjusted HR for postoperative TKI-ICI exposure** | **95% CI** | ***p*** |
| --- | --- | --- | --- | --- | --- |
| MT-HCC | 110 | 49 | 0.13 | 0.05–0.34 | <0.001 |
| non-MT-HCC | 228 | 63 | 1.08 | 0.57–2.02 | 0.822 |

**Abbreviations:** HR, hazard ratio; CI, confidence interval; MT-HCC, MVI+/TLSs+ hepatocellular carcinoma; TKI, tyrosine kinase inhibitor; ICI, immune checkpoint inhibitor.

**Note:** Stratified Cox models were adjusted for age, sex, AFP level (>400 ng/mL), BCLC stage, and tumor size.
